# In silico identification of single nucleotide variations at CpG sites regulating CpG island existence and size

**DOI:** 10.1038/s41598-022-05198-8

**Published:** 2022-03-04

**Authors:** Nivas Shyamala, Chaitra Lava Kongettira, Kaushik Puranam, Keerthi Kupsal, Ramanjaneyulu Kummari, Chiranjeevi Padala, Surekha Rani Hanumanth

**Affiliations:** 1 Present Address: Department of Genetics and Biotechnology, University College of Science, Osmania University, Hyderabad, 500007 Telangana State India; 2 Department of Biochemistry, School of Life Sciences, University of Hyderabad, Hyderabad, 500046 Telangana State India

**Keywords:** Cancer, Computational biology and bioinformatics, Genetics, Molecular biology, Cardiology, Diseases, Endocrinology

## Abstract

Genetic and epigenetic modifications of genes involved in the key regulatory pathways play a significant role in the pathophysiology and progression of multifactorial diseases. The present study is an attempt to identify single nucleotide variations (SNVs) at CpG sites of promoters of *ACAT1*, *APOB*, *APOE*, *CYBA*, *FAS*, *FLT1*, *KSR2*, *LDLR*, *MMP9*, *PCSK9*, *PHOX2A*, *REST*, *SH2B3*, *SORT1* and *TIMP1* genes influencing CpG island (CGI) existence and size associated with the pathophysiology of Diabetes mellitus, Coronary artery disease and Cancers. Promoter sequences located between −2000 to + 2000 bp were retrieved from the EPDnew database and predicted the CpG island using MethPrimer. Further, SNVs at CpG sites were accessed from NCBI, Ensembl while transcription factor (TF) binding sites were accessed using AliBaba2.1. CGI existence and size were determined for each SNV at CpG site with respect to wild type and variant allele by MethPrimer. A total of 200 SNVs at CpG sites were analyzed from the promoters of *ACAT1*, *APOB*, *APOE*, *CYBA*, *FAS*, *FLT1*, *KSR2*, *LDLR*, *MMP9*, *PCSK9*, *PHOX2A*, *REST*, *SH2B3*, *SORT1* and *TIMP1* genes. Of these, only 17 (8.5%) SNVs were found to influence the loss of CGI while 70 (35%) SNVs were found to reduce the size of CGI. It has also been found that 59% (10) of CGI abolishing SNVs are showing differences in binding of TFs. The findings of the study suggest that the candidate SNVs at CpG sites regulating CGI existence and size might influence the DNA methylation status and expression of genes involved in molecular pathways associated with several diseases. The insights of the present study may pave the way for new experimental studies to undertake challenges in DNA methylation, gene expression and protein assays.

## Introduction

Multifactorial diseases like Diabetes mellitus (DM), Coronary artery disease (CAD) and Cancers are the top leading causes of death worldwide^1^. Globally, understanding of underlying mechanisms and prevention of these diseases with different strategies are potential challenges for researchers in medicine^2^. These diseases are influenced by common risk factors such as family history, smoking, obesity, insufficient physical activity, etc^3^. Studies suggest that besides these conventional risk factors, genetic and epigenetic modifications of certain genes also play a significant role in pathophysiology and progression of these diseases^4–6^.

Evidences suggest that epigenetic modifications regulate the genome structure and expression pattern of genes^7,8^. These mechanisms include DNA methylation, histone modification and non-coding RNAs regulation, which can be inherited from one generation to the next^9^. DNA methylation is a common molecular alteration at CpG sites of DNA sequence which is influenced by genetic and environmental factors. DNA methylation in various cell types regulate the expression of genes and shows an association with the pathophysiology of diseases^10–13^.

DNA methylation at CpG sites is an enzymatic reaction catalysed and maintained by DNA methyltransferase (DNMT) family in particular DNMT3A, 3B and DNMT1^14^. DNMTs convert cytosine to 5-methylcytosine by adding methyl group at CpG dinucleotide sites of CpG islands (CGIs). CGIs are typically located at the regulatory regions, predominantly in promoters and are 500-1500 bp long^15,16^. Commonly, transcriptional activity of promoter depends on the binding efficiency of RNA polymerase II and transcription factors (TF) to the core promoter^17^. Studies suggested that the methylation of cytosines in a promoter DNA suppresses the rate of transcription, reduces the mRNA copy number and ultimately affects the protein synthesis^18–20^.

Initially, genes under the study *ACAT1*^21,22^, *APOB*^23,24^, *APOE*^25–27^, *CYBA*^28,29^, *FAS*^30,31^, *FLT1*^32,33^, *KSR2*^34^, *LDLR*^24,35^, *MMP9*^36,37^, *PCSK9*^13,38,39^, *PHOX2A*^40–42^, *REST*^43,44^, *SH2B3*^45–47^, *SORT1*^48–50^ and *TIMP1*^51,52^ were selected which were found to be involved in several key regulatory pathways associated with the pathology of DM, CAD and Cancers (Supplementary Table 1). These genes and gene products enormously involve in various pathways: *ACAT1*, *PCSK9 & SORT1* in cholesterol homeostasis; *APOB*, *APOE & LDLR* in lipid metabolism; *CYBA*, *KSR2 & PHOX2A* in oxidative stress; *FAS*, *REST* & *SORT1* in apoptosis; *FLT1 & SH2B3* in inflammation and angiogenesis; *MMP9 & TIMP1* in maintenance of extracellular matrix and vascular smooth muscle cells.

Studies suggest that the single nucleotide variations (SNVs) located at promoter, exonic & intronic regions of these genes regulate the expression, alternative splicing of mRNA, structural conformation of proteins, etc^28,30,31,36,53^. Moreover, these genes were found to have genome-wide significant loci for risk of multifactorial diseases in various populations. In addition, epigenetic studies have suggested that the DNA methylation of *ACAT1*^54^, *APOB*^55^, *APOE*^19^, *CYBA*^6^, *FAS*^20^, *FLT1*^56^, *LDLR*^57^, *MMP9*^58^, *PCSK9*^13,59^, *REST*^60^, *SH2B3*^61^, *SORT1*^62^ and *TIMP1*^63^ genes play a substantial role in regulation of gene expression.

There are few reports published to show the tangible impact of SNVs at CpG sites on CpG island existence or size in genes influencing the pathophysiology of various diseases^64–66^. A genome-wide CpG SNP identification study revealed that CpG SNPs are significantly associated with the Cancers^64^. Furthermore, GWAS datasets on DM and CAD have identified novel functional SNPs at CpG sites which affect the expression and function of genes via epigenetic regulations^65^. Experimental studies on O6-methylguanine-DNA methyltransferase (*MGMT*) gene rs16906252 and *RAD50* gene DNase I hypersensitive sites (*RHS*) 7 region rs2240032 polymorphisms suggested that SNPs at CpG sites can influence the DNA methylation at promoter regions, transcription factors binding at enhancer or silencer region and miRNA binding at 3’UTR region^67–70^. The SNVs at CpG sites might modulate the existence and size of CpG islands at the promoter region; altering the methylation patterns and binding of transcription factors which ultimately affect the gene activation or silencing or expression^64,65^. Therefore, studies are warranted to identify SNVs at CpG sites regulating CpG island existence & size and their consequent effects on DNA methylation and gene expression.

Hence, the present study is an attempt to identify candidate SNVs at CpG sites in promoter region of *ACAT1*, *APOB*, *APOE*, *CYBA*, *FAS*, *FLT1*, *KSR2*, *LDLR*, *MMP9*, *PCSK9*, *PHOX2A*, *REST*, *SH2B3*, *SORT1* and *TIMP1* genes regulating the existence and size of CpG islands.

## Materials and methods

### Study design

**Figure 1:**
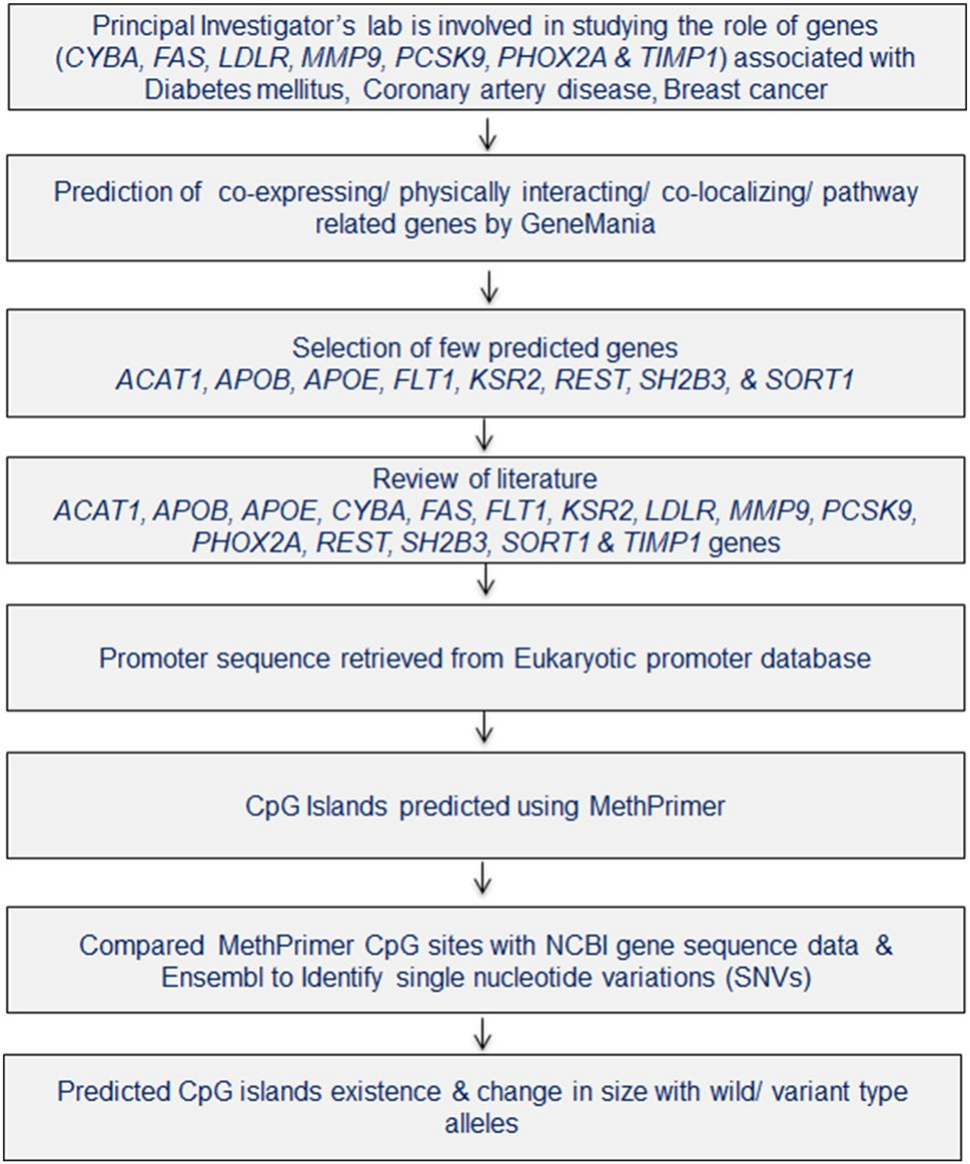
Schematic representation of study design.

The detailed study design is presented in Fig. 1.

### Literature search and databases

We have conducted a comprehensive electronic search to browse genes under study, SNVs data and their respective literature using following data bases: National Library of Medicine (https://www.nlm.nih.gov/), National Center for Biotechnology Information (NCBI) (https://www.ncbi.nlm.nih.gov/), PubMed (https://pubmed.ncbi.nlm.nih.gov/), dbSNP (https://www.ncbi.nlm.nih.gov/snp/), Cancer Genetics Web (http://www.cancerindex.org/geneweb/), Google scholar (https://scholar.google.com/), GeneCards: the human gene database (https://www.genecards.org/). The search was limited to key words *‘ACAT1’*, *‘APOB’*, *‘APOE’*, *‘CYBA’*, *‘FAS’*, *‘FLT1’*, *‘KSR2’*, *‘LDLR’*, *‘MMP9’*, *‘PCSK9’*, *‘PHOX2A’*, *‘REST’*, *‘SH2B3’*, *‘SORT1’ ‘TIMP1’*, polymorphisms, genetic variations, CpG islands, DNA methylation, Diabetes mellitus, Coronary artery disease and Cancer.

### Promoter sequence retrieval

Promoter sequences located between − 2000 to + 2000 bp were retrieved from Eukaryotic promoter database (EPD) new to check the CpG island status of genes under the study. EPD new allows access to several databases of experimentally validated promoters and published articles of model organisms. EPDnew contains 4806 promoters from various species like *Homo sapiens*, *Mus musculus*, *Caenorhabditis elegans*, *Drosophila melanogaster*, *Arabidopsis thaliana*, *Saccharomyces cerevisiae*, etc.^71^.

### Prediction of CpG Islands

CpG islands (CGIs) in promoter sequence of genes under the study were predicted using MethPrimer v1.1 beta. CGI existence and size were determined for each single nucleotide variation at CpG site with respect to wild type and variant allele. MethPrimer predicts potential CGIs in the input promoter DNA sequence and designs sequence specific primers for Methylation-Specific PCR and Bisulfite-Sequencing PCR. The output results are presented in graphical view for predicted CpG island and in text format for PCR primers^72^. The criteria used for gain and loss of CGI prediction is Island size > 100bp, GC percent > 50.0, ratio of Obs/Exp no of CpG dinucleotides > 0.60^73^.

### Selection of SNVs at CpG sites

CpG sites were identified from the results of MethPrimer and the SNVs at CpG sites were accessed from National Center for Biotechnology Information (NCBI) and Ensembl. NCBI and Ensembl are widely used genome browsers in global scientific community. The browsers were developed with the data of genomic regions, genes, gene sequence, genetic variations, phenotypes, etc. The tools visualize DNA sequence and their respective annotated genetic variations to identify the SNVs at CpG sites in CpG islands^74,75^.

### Transcription factor binding site prediction

AliBaba2.1 tool was used for the prediction of transcription factor binding sites in wild type and variant alleles of SNVs at CpG sites. It is an online tool to identify transcription factors and their respective binding sites for the input DNA sequence by constructing matrices on the fly from TRANSFAC 4.0 sites. AliBaba tool has significantly higher sensitivity and sensitivity/specificity ratio than other current approaches^76^.

### Co-expression prediction

*APOE,* *CYBA, FAS, LDLR, MMP9, PCSK9, PHOX2A, SH2B3* and *TIMP1* genes were analysed to know the other co-expressing, physically interacting, co-localizing and key biological pathway related genes using GeneMANIA. GeneMANIA is a potent database of almost 2300 networks with 600 million interactions covering upto 164,000 genes in model organisms and provide genomic, proteomic, and gene function data. It is an effective approach to predict the function of input single gene/ multiple gene queries physically interacting proteins, co-expressing and co-localizing genes, genetic interactions, shared protein domains and pathways^77,78^.

Layouts generated by GeneMANIA web server have nodes and edges. Nodes represent gene and its products, while edges represent co-expression interaction and weight of each edge implies the evidence of co-functionality data source.

### Gene ontology enrichment analysis

Gene ontology (GO) enrichment analysis of genes (*ACAT1*, *APOB*, *APOE*, *CYBA*, *FAS*, *FLT1*, *KSR2*, *LDLR*, *MMP9*, *PCSK9*, *PHOX2A*, *REST*, *SH2B3*, *SORT1*, *TIMP1*) was performed using Database for Annotation, Visualization and Integrated Discovery (DAVID) v6.8 online tool (https://david.ncifcrf.gov/home.jsp). The GO terms were classified into three categories: biological process (BP), cellular component (CC) and molecular function (MF) with significant p value of <0.05. Further, GO term enrichment analysis was used to annotate the disease class and functional clustering of genes under the study.

## Results

**Figure 2:**
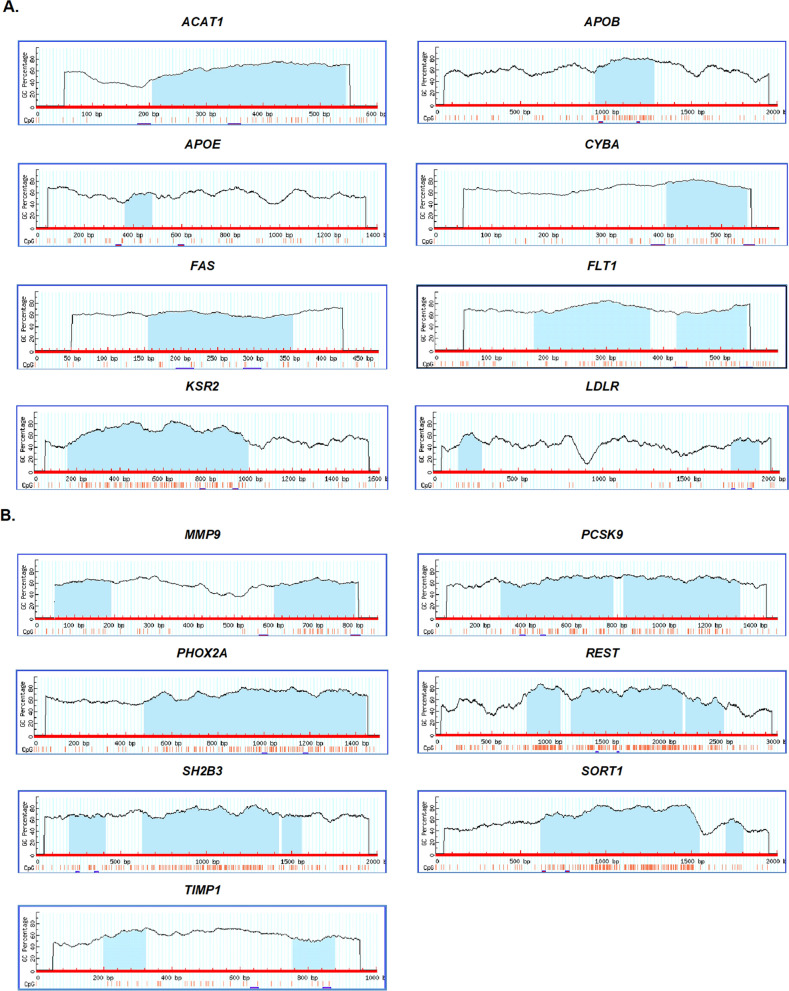
CpG islands prediction in promoter sequence of genes. (**A**) *ACAT1*, *APOB*, *APOE*, *CYBA*, *FAS*, *FLT1*, *KSR2*, *LDLR*. (**B**) *MMP9*, *PCSK9*, *PHOX2A*, *REST*, *SH2B3*, *SORT1*, *TIMP1*. The figure consists:  input sequence to predict the CpG islands and to design bisulfite/ methylation specific PCR primers,  CpG island region.

Promoter sequence of *ACAT1*, *APOB*, *APOE*, *CYBA*, *FAS*, *FLT1*, *KSR2*, *LDLR*, *MMP9*, *PCSK9*, *PHOX2A*, *REST*, *SH2B3*, *SORT1* and *TIMP1* genes were analysed for the prediction of CpG islands and have observed CpG islands for all the genes (Fig. 2A, B). Further, the existence and sizes of CGI for wild type and variant alleles of all the CpG SNVs were analyzed. In addition, transcription factors binding to both the wild type and variant alleles of CpG SNVs abolishing CGI were predicted.

**Figure 3:**
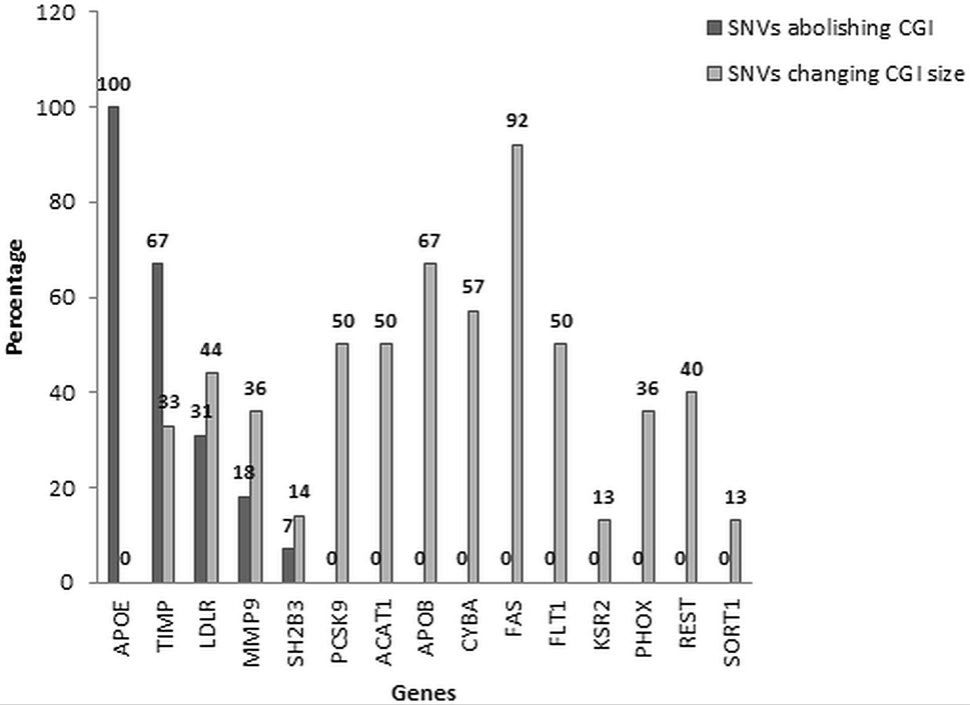
Single nucleotide variations showing influence on CGIs status & size for *ACAT1*, *APOB*, *APOE*, *CYBA*, *FAS*, *FLT1*, *KSR2*, *LDLR*, *MMP9*, *PCSK9*, *PHOX2A*, *REST*, *SH2B3*, *SORT1* and *TIMP1 *genes.

A total of 200 SNVs at CpG sites were studied for *ACAT1* (10), *APOB* (3), *APOE* (1), *CYBA* (7), *FAS* (12), *FLT1* (6), *KSR2* (31), *LDLR* (16), *MMP9* (28), *PCSK9* (8), *PHOX2A* (22), *REST* (5), *SH2B3* (29), *SORT1* (16) and *TIMP1* (6) genes. Of these, 17 (8.5%) candidate SNVs abolished the CpG islands existence and 70 (35%) SNVs potentially decreased the CpG islands size in various genes (Table 1).The percentage of abolished CGIs and change in size of CGIs of all genes are represented in Table 1 and Fig. 3.

**Table 1:** Single nucleotide variations (SNVs) at CpG sites associated with loss or change in the size of CpG island.

S. No.	CpG island and size (bp)	Single nucleotide variations (SNVs) (rs number; variation)	CpG coordinates on chromosome	CpG island status with		Change in CpG island size (bp)
				Wild type allele	Variant allele	
**Gene**	**Acetyl-Coenzyme A acetyltransferase 1 (*ACAT1*)**					
1	Island;341	rs539426263;C/A*	chr11:108121278	Present	Present	339
2		rs376263677;G/C	chr11:108121289	Present	Present	341
3		rs376263677;G/T*		Present	Present	339
4		rs979540931;C > G*	chr11:108121307	Present	Present	339
5		rs551761017;C > A*	chr11:108121313	Present	Present	339
6		rs1191223847;G > A*	chr11:108121314	Present	Present	339
7		rs1294688280;C > T	chr11:108121367–108121378	Present	Present	341
8		rs1294688280;G > A		Present	Present	341
9		rs1246409549;C > T	chr11:108121403	Present	Present	341
10		rs1197006182;G > A	chr11:108121404	Present	Present	341
**Gene**	**Apolipoprotein B (*APOB*)**					
11	Island;344	rs745633995;G/A*	chr2:21044088	Present	Present	340
12		rs956977643;C/T*	chr2:21044082	Present	Present	343
13		rs973345426;C/A	chr2:21044076	Present	Present	344
**Gene**	**Apolipoprotein E (*APOE*)**					
14	Island;112	rs769448;C/T**	chr19:44906322	Present	Abolished	0
**Gene**	**Cytochrome b-245 alpha chain (*CYBA*)**					
15	Island;136	rs1021215371;C/T*	chr16:88651087	Present	Present	135
16		rs544939582;G/A*	chr16:88651070	Present	Present	135
17		rs756019435;C/T*	chr16:88651047	Present	Present	135
18		rs376510042;G/T*	chr16:88651064	Present	Present	135
19		rs756019435;C/T	chr16:88651047	Present	Present	136
20		rs750384376;G/A	chr16:88651046	Present	Present	136
21		rs373406027;G/A	chr16:88651027	Present	Present	136
**Gene**	**Factor associated suicide death receptor (*FAS*)**					
22	Island 1;199	rs752145197;G/C*	chr10:88990538	Present	Present	190
23		rs755644207;C/T*	chr10:88990539	Present	Present	177
24		rs886047456;G/A*	chr10:88990540	Present	Present	191
25		rs777366435;C/A*	chr10:88990541	Present	Present	190
26		rs533623533;G/A*	chr10:88990542	Present	Present	191
27		rs9658677;G/A	chr10:88990582	Present	Present	199
28		rs902017811;C/A*	chr10:88990595	Present	Present	128
29		rs1021894100;C/T*	chr10:88990642	Present	Present	128
30		rs769222279;G/C*	chr10:88990643	Present	Present	128
31		rs777296029;C/A*	chr10:88990656	Present	Present	128
32		rs904814296;G/C*	chr10:88990657	Present	Present	128
33		rs557366318;G/A*	chr10:88990715	Present	Present	184
**Gene**	**Fms related tyrosine kinase 1 (*FLT1*)**					
34	Island 1;211	rs935059277;G/C	chr13:28495711	Present	Present	211
35		rs61763160;C/T*	chr13:28495681	Present	Present	199
36		rs1024357361;G/A*	chr13:28495655	Present	Present	198
37		rs779832391;G/A*	chr13:28495524	Present	Present	188
38	Island 2;204	rs1028125144;C/G	chr13:28495300	Present	Present	188
39		rs998030865;G/T	chr13:28495276	Present	Present	188
**Gene**	**Kinase suppressor of ras 2 (*KSR2*)**					
40	Island;838	rs73408418;C/T*	chr12:117969559	Present	Present	803
41		rs962883023;G/A*	chr12:117969543	Present	Present	804
42		rs1010334504;G/C	chr12:117969521	Present	Present	838
43		rs891447546;G/T/A—T	chr12:117969518	Present	Present	838
44		rs552191962;G/C	chr12:117969510	Present	Present	838
45		rs182966035;G/A	chr12:117969500	Present	Present	838
46		rs939897252;CCCAGCCGGAGCGCACCTGCT/-*	chr12:117969450–117969478	Present	Present	817
47		rs1011133176;C/T	chr12:117969464	Present	Present	838
48		rs114278232;G/A	chr12:117969418	Present	Present	838
49		rs528230001;C/G	chr12:117969394	Present	Present	838
50		rs7300907;G/C/A—C	chr12:117969393	Present	Present	838
51		rs1034361818;G/C	chr12:117969386	Present	Present	838
52		rs931680247;C/A	chr12:117969367	Present	Present	838
53		rs898886083;G/C	chr12:117969341	Present	Present	838
54		rs545819605;C/T	chr12:117969330	Present	Present	838
55		rs971514425;G/A	chr12:117969329	Present	Present	838
56		rs908447922;TCCCCCGCCGCCCC/-*	chr12:117969312–117969327	Present	Present	824
57		rs927580374;G/A	chr12:117969310	Present	Present	838
58		rs968768275;C/T	chr12:117969289	Present	Present	838
59		rs1022089500;C/T	chr12:117969287	Present	Present	838
60		rs954962287;G/C	chr12:117969273	Present	Present	838
61		rs956144219;C/G	chr12:117969268	Present	Present	838
62		rs890348830;G/A	chr12:117969244	Present	Present	838
63		rs557703958;G/T/C T	chr12:117969236	Present	Present	838
64		rs999829657;G/T	chr12:117969228	Present	Present	838
65		rs886214687;G/A	chr12:117969152	Present	Present	838
66		rs1057218279;C/A	chr12:117969151	Present	Present	838
67		rs535742283;C/T	chr12:117969140	Present	Present	838
68		rs534893029;G/T/A—T	chr12:117969130	Present	Present	838
69		rs974051469;C/T	chr12:117969128	Present	Present	838
70		rs980137500;G/C	chr12:117969116	Present	Present	838
**Gene**	**Low density lipoprotein receptor (*LDLR*)**					
71	Island 1;138	rs531870546;C/G	chr19:11087615	Present	Present	138
72		rs543676881;G/A/T *	chr19:11087616	Present	Present	136
73		rs1026272027;G/T**	chr19:11087638	Present	Abolished	0
74		rs887608252;C/T**	chr19:11087645	Present	Abolished	0
75		rs1006494933;G/A**	chr19:11087646	Present	Abolished	0
76		rs532491368;G/A**	chr19:11087670	Present	Abolished	0
77		rs1024897634;C/T**	chr19:11087677	Present	Abolished	0
78		rs1038399041;C/T*	chr19:11087733	Present	Present	108
79		rs899331076;G/A*	chr19:11087734	Present	Present	108
80		rs371798074;C/T*	chr19:11087737	Present	Present	108
81		rs1046779346;G/C	chr19:11087738	Present	Present	138
82	Island 2;167	rs574713917;C/G	chr19:11089227	Present	Present	167
83		rs17249134;G/T	chr19:11089281	Present	Present	167
84		rs17249141;C/T*	chr19:11089332	Present	Present	152
85		rs549995837;C/T*	chr19:11089343	Present	Present	152
86		rs182017676;C/A*	chr19:11089347	Present	Present	152
**Gene**	**Matrix metalloproteinase 9 (*MMP9*)**					
87	Island 1;172	rs139620474;C/A/T—A** or C/A/T—T**	chr20:46009878	Present	Abolished	0
88		rs370018925;C/T**	chr20:46009908	Present	Abolished	0
89		rs201069991;G/A**	chr20:46009909	Present	Abolished	0
90		rs1014494202;C/T**	chr20:46009936	Present	Abolished	0
91		rs146719297;G/A**	chr20:46009937	Present	Abolished	0
92		rs200849957;C/G/T—G or C/G/T—T	chr20:46009970	Present	Present	172
93		rs1805089;G/A	chr20:46009971	Present	Present	172
94		rs1023660861;C/T	chr20:46009976	Present	Present	172
95		rs143695450;G/A/T—A or T	chr20:46009977	Present	Present	172
96		rs45482493;C/T	chr20:46009991	Present	Present	172
97		rs377251829;C/A	chr20:46010010	Present	Present	172
98		rs140352541;G/T	chr20:46010020	Present	Present	172
99	Island 2;205	rs762336901;C/T*	chr20:46010433	Present	Present	137
100		rs765973004;C/G*	chr20:46010475	Present	Present	135
101		rs756724622;C/G*	chr20:46010497	Present	Present	134
102		rs749347450;C/T*	chr20:46010509	Present	Present	134
103		rs200637345;C/T*	chr20:46010511	Present	Present	134
104		rs757458476;C/T*	chr20:46010515	Present	Present	150
105		rs745724816;G/-*	chr20:46010529	Present	Present	149
106		rs776477347;G/A*	chr20:46010539	Present	Present	150
107		rs201902138;C/G/T—G or C/G/T—T*	chr20:46010558	Present	Present	149
108		rs767959655;G/A*	chr20:46010561	Present	Present	149
109		rs753889026;C/A	chr20:46010569	Present	Present	205
110		rs777580909;G/A	chr20:46010628	Present	Present	205
111		rs202214757;C/A	chr20:46010629	Present	Present	205
112		rs183834856;G/A	chr20:46010630	Present	Present	205
113		rs984503896;C/A	chr20:46010639	Present	Present	205
114		rs201044639;G/A	chr20:46010640	Present	Present	205
**Gene**	**Proprotein convertase subtilisin/kexin type 9 (*PCSK9*)**					
115	Island;491	rs911797629;C > T*	chr1:55039338	Present	Present	464
116		rs987969811;G > A*	chr1:55039389	Present	Present	464
117		rs371053631;C/T*	chr1:55039390	Present	Present	464
118		rs978397913;G/A*	chr1:55039391	Present	Present	464
119		rs865997599;C/T	chr1:55039416	Present	Present	491
120		rs887437926;G/T	chr1:55039452	Present	Present	491
121		rs188274059;C/A/T	chr1:55039516	Present	Present	491
122		rs745962158;G/A	chr1:55039517	Present	Present	491
**Gene**	***Paired like homeobox 2a (PHOX2A)***					
123	Island;964	rs946255361;G/A*	chr11:72244638	Present	Present	880
124		rs985554082;C/G	chr11:72244600	Present	Present	964
125		rs565201625;C/A*	chr11:72244597	Present	Present	879
126		rs545309058;G/A*	chr11:72244596	Present	Present	880
127		rs919731208;G/T*	chr11:72244574	Present	Present	880
128		rs973079104;G/C	chr11:72244555	Present	Present	964
129		rs904705949;C/G/A -G	chr11:72244511	Present	Present	964
130		rs1021763886;G/A	chr11:72244510	Present	Present	964
131		rs1010395824;C/G	chr11:72244507	Present	Present	964
132		rs950416969;G/C	chr11:72244371	Present	Present	964
133		rs959032571;G/C*	chr11:72244355	Present	Split	315;641
134		rs553752383;G/A*	chr11:72244322	Present	Split	390;571
135		rs1021105224;G/A*	chr11:72244319	Present	Split	390;571
136		rs1019884836;G/A*	chr11:72244305	Present	Split	390;571
137		rs889804293;G/C	chr11:72244293	Present	Present	964
138		rs917708636;C/T	chr11:72244248	Present	Present	964
139		rs937911897;C/T	chr11:72244236	Present	Present	964
140		rs987854333;C/G	chr11:72244197	Present	Present	964
141		rs992203984;G/A	chr11:72244196	Present	Present	964
142		rs956196630;G/T	chr11:72244194	Present	Present	964
143		rs1019771178;C/T	chr11:72244193	Present	Present	964
144		rs1008498233;G/T	chr11:72244187	Present	Present	964
**Gene**	**RE1 silencing transcription factor (*REST*)**					
145	Island;298	rs964635804;G/A*	chr4:56907734	Present	Present	291
146		rs982281493;G/C	chr4:56907790	Present	Present	298
147		rs928222537;G/C	chr4:56907803	Present	Present	298
148		rs938247687;G/A	chr4:56907809	Present	Present	298
149		rs1047872828;G/GGCGGT*	chr4:56907870–56907874	Present	Present	304
Gene	**SH2B adaptor protein 3 (*SH2B3*)**					
150	Island 1;214	rs960136772;G/A*	chr12:111405136	Present	Present	150
151		rs538445017;C/T**	chr12:111405235	Present	Abolished	0
152		rs922413124;G/A**	chr12:111405236	Present	Abolished	0
153		rs995735060;C/A*	chr12:111405248	Present	Present	114
154		rs574117302;C/T	chr12:111405270	Present	Present	214
155	Island 2;796	rs542650199;C/A/G—A or C/A/G—G*	chr12:111405555	Present	Present	778/754
156		rs1028968561;C/T*	chr12:111405609	Present	Present	778
157		rs1042427838;C/A	chr12:111405693	Present	Present	796
158		rs763506765;G/C	chr12:111405694	Present	Present	796
159		rs899785538;C/A	chr12:111405709	Present	Present	796
160		rs75390213;G/A	chr12:111405712	Present	Present	796
161		rs943838180;G/A	chr12:111405728	Present	Present	796
162		rs982567306;G/T	chr12:111405743	Present	Present	796
163		rs1015319598;C/A	chr12:111405750	Present	Present	796
164		rs1029498594;G/A	chr12:111405764	Present	Present	796
165		rs974278790;C/A/T—A or C/A/T—T	chr12:111405774	Present	Present	796
166		rs532367698;G/T	chr12:111405775	Present	Present	796
167		rs1013689151;G/A	chr12:111405795	Present	Present	796
168		rs917942737;G/C	chr12:111405807	Present	Present	796
169		rs566012237;C/T	chr12:111405823	Present	Present	796
170		rs1005740439;G/C	chr12:111405854	Present	Present	796
171		rs1054248299;C/T	chr12:111405879	Present	Present	796
172		rs890806829;C/T	chr12:111405889	Present	Present	796
173		rs1015267150;G/T	chr12:111405900	Present	Present	796
174		rs962487794;C/T	chr12:111405903	Present	Present	796
175		rs868119397;G/C/T—C or G/C/T—T	chr12:111405908	Present	Present	796
176		rs1033875297;C/T	chr12:111405929	Present	Present	796
177		rs959781377;G/C	chr12:111405930	Present	Present	796
178		rs992435554;G/A	chr12:111405940	Present	Present	796
**Gene**	**Sortilin 1 *(SORT1)***					
179	Island;931	rs915825764;C/T*	chr1:109398261	Present	Present	928
180		rs968169903;C/T	chr1:109398201	Present	Present	931
181		rs112431410;C/G	chr1:109398185	Present	Present	931
182		rs1056848876;C/T/G—T	chr1:109398179	Present	Present	931
183		rs1003657108;G/C	chr1:109398178	Present	Present	931
184		rs1037052612;G/A	chr1:109398159	Present	Present	931
185		rs188539890;C/T	chr1:109398133	Present	Present	931
186		rs544729829;G/T	chr1:109398113	Present	Present	931
187		rs992705461;C/T	chr1:109398085	Present	Present	931
188		rs574878989;C/G*	chr1:109398085–109398089	Present	Present	932
189		rs978471974;G/C	chr1:109398069	Present	Present	931
190		rs1043020951;C/G	chr1:109398068	Present	Present	931
191		rs1022467277;C/G	chr1:109398031	Present	Present	931
192		rs1031024794;C/T	chr1:109398005	Present	Present	931
193		rs1001269821;G/C	chr1:109397996	Present	Present	931
194		rs903970476;G/C	chr1:109397969	Present	Present	931
**Gene**	**Tissue inhibitor of metalloproteinase 1 (*TIMP1*)**					
195	Island 1;126	rs779329701;G/A**	chrX:47582148	Present	Abolished	0
196		rs993047389;G/A**	chrX:47582175	Present	Abolished	0
197		rs376386551;C/T**	chrX:47582232	Present	Abolished	0
198		rs926004266;G/A**	chrX:47582233	Present	Abolished	0
199	Island 2;125	rs895934083;G/A*	chrX:47582749	Present	Present	105
200		rs936052046;C/A/T—A or C/A/T—T*	chrX:47582798	Present	Present	109

**indicates the SNVs abolish CpG island, *indicates the SNVs change CpG island size; rs:reference sequence

### CpG SNVs abolishing and reducing sizes of CGI

*APOE* gene has a single SNV rs769448 at CpG site, its variant allele has lost the entire 112 bp CGI. Among the 16 CpG SNVs studied in 2 CGIs (island 1:138 bp, island 2:167 bp) of *LDLR* gene, 5 SNVs (rs1026272027, rs887608252, rs1006494933, rs532491368, rs1024897634) have abolished the entire CGI whereas 7 SNVs have shown a 2-30 bp reduction in CGI size.

In *SH2B3* gene, 29 CpG SNVs were studied in 2 CpG islands (island 1:214 bp; island 2:796 bp), out of which 2 SNVs (rs538445017, rs922413124) in the first CGI has abolished the entire CGI. Whereas, remaining 3 SNVs in the first CGI and the other 2 SNVs in second CGI have shown a 18–100 bp decrease in the size of CGI.

Amongst the 28 CpG SNVs selected in 2 CpG islands (island 1:172 bp; island 2:205 bp) of *MMP9* gene, 5 SNVs (rs139620474, rs370018925, rs201069991, rs1014494202, rs146719297) in the first CGI have abolished the entire CGI, while 10 SNVs in the second CpG island have reduced 55-71 bp in their sizes.

In *TIMP1* gene, 6 SNVs were analyzed in 2 CpG islands (island 1:126 bp; island 2:125 bp), the results revealed that 4 SNVs (rs779329701, rs993047389, rs376386551, rs926004266) in the first CGI have abolished the entire CGI, whereas the remaining 2 SNVs in the second CpG island have shown a 16-20 bp reduction in their CGI size.

Further, the CpG site SNVs 5 in *ACAT1*, 2 in *APOB*, 4 in *CYBA*, 11 in *FAS*, 5 in *FLT1,* 4 in *KSR2*, 4 in *PCSK9*, 8 in *PHOX2A*, 2 in *REST* and 2 in *SORT1* are reducing the CGI sizes ranging from 1-85 bp.

### Transcription factor binding site analysis

SNVs at CpG sites abolishing the CGIs of *LDLR*, *MMP9*, *SH2B3*, *TIMP1* and *APOE* *1* genes were analysed to predict the difference in binding of transcription factors (TF) at the site of variation. As represented in Table 2, we have observed that SNVs 4 in *LDLR*, 2 in *MMP9*, 1 in *SH2B*3, 2 in *TIMP1* and 1 in *APOE* genes have shown a difference in binding of TFs.

**Table 2:** Transcription factors associated with the single nucleotide variations (SNVs) abolishing CGIs.

Gene	Single nucleotide variations (rs number; variation)	Transcription factors	
		Wild type allele	Variant allele
Low density lipoprotein receptor (*LDLR*)	rs1026272027,G/T*	C/EBPapl	C/EBPbet
	rs887608252,C/T*	No TF	C/EBPapl
	rs1006494933,G/A*	No TF	GATA-1, Oct-1
	rs532491368,G/A	No TF	No TF
	rs1024897634,C/T*	No TF	Oct-1
Matrix metalloproteinase 9 (*MMP9*)	rs139620474,C/A/T –A or –T	No TF	No TF
	rs370018925,C/T*	No TF	Sp1
	rs201069991,G/A	No TF	No TF
	rs1014494202,C/T*	Sp1	Sp1, BRF-1
	rs146719297, G/A	Sp1	Sp1
SH2B adaptor protein 3 (*SH2B3*)	rs538445017,C/T	Tra-1	Tra-1
	rs922413124,G/A*	Sp-1	No TF
Tissue inhibitor of metalloproteinase 1 (*TIMP1*)	rs779329701,G/A*	Egr-1	NF-1
	rs993047389,G/A	Sp1	Sp1
	rs376386551,C/T*	Sp1	N-Myc
	rs926004266,G/A	Sp1	Sp1
Apolipoprotein E (*APOE*)	rs769448, C/T*	Sp1	No TF

*change in transcription factor binding; No TF: No transcription factor

To the 4 SNVs of *LDLR* gene that abolished CGI, TFs binding site prediction has shown that rs1026272027 wild type allele has a binding site for C/EBPapl and variant allele has a binding site for C/EBPbet. For rs887608252,C/T, rs1006494933,G/A and rs1024897634,C/T SNVs, there were no TF binding sites for their wild type alleles, but their variant alleles have binding sites for C/EBPapl, GATA-1 & Oct-1 and Oct-1 TFs respectively.

Likewise, 2 SNVs abolishing CGIs in *MMP9* gene have shown the difference in binding of TFs, rs370018925 wild type allele has no binding site for any TF whereas variant allele is bound by Sp1 transcription factor. Though the rs1014494202 has Sp1 binding site for wild type allele, variant allele has an additional binding site for BRF-1 transcription factor.

For rs922413124 in *SH2B3* gene, there was a binding site for Sp1 in wild type allele, but it is abolished in variant allele. Similarly, *APOE* rs769448 has binding site for Sp1 transcription factor but its variant allele is lacking a site for binding of any transcription factor.

Furthermore, 2 SNVs that abolished CGIs in *TIMP1* gene has shown that the wild type alleles of rs779329701 and rs376386551 has binding sites for Egr-1 and Sp1 transcription factors while variant alleles have binding sites for NF-1 and N-Myc transcription factors respectively.

### Co-expression analysis

**Figure 4:**
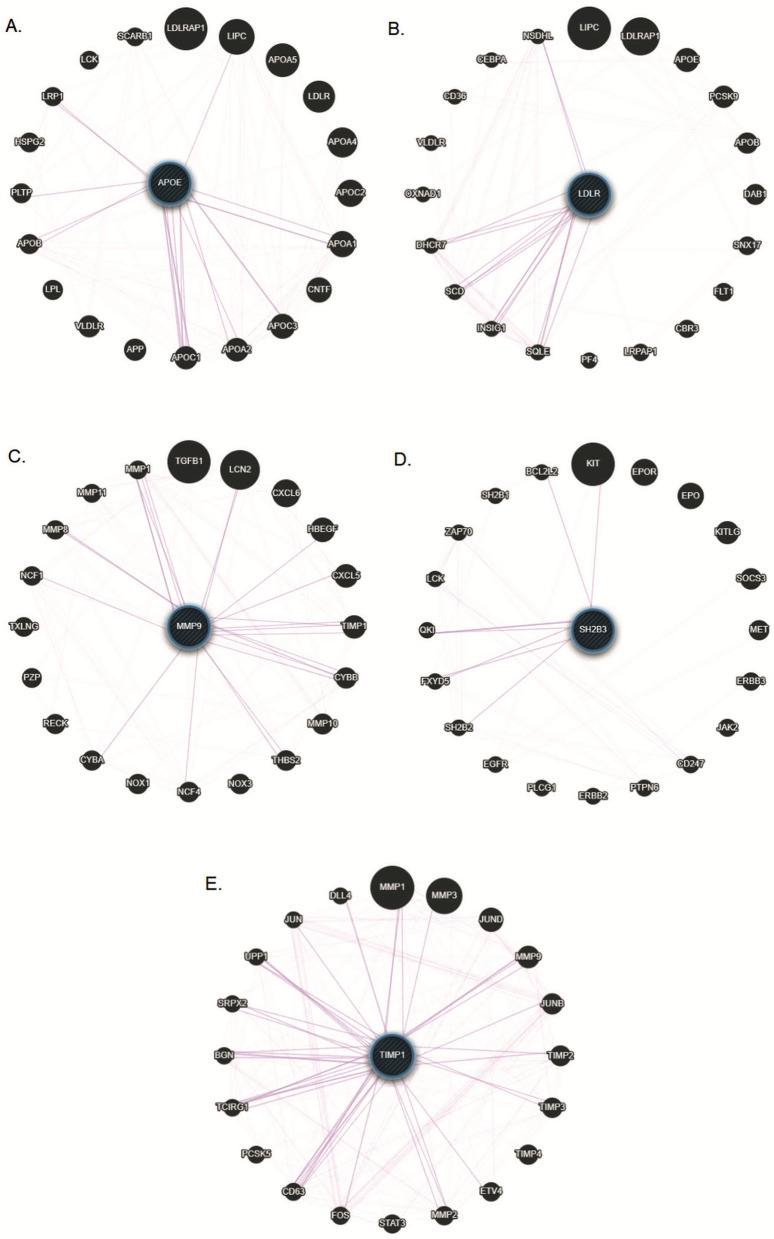
Concentric bipartites by GeneMANIA represents Co-expression networks of *A.APOE B.LDLR C.MMP9 D.SH2B3 E.TIMP1* genes.

**Figure 5:**
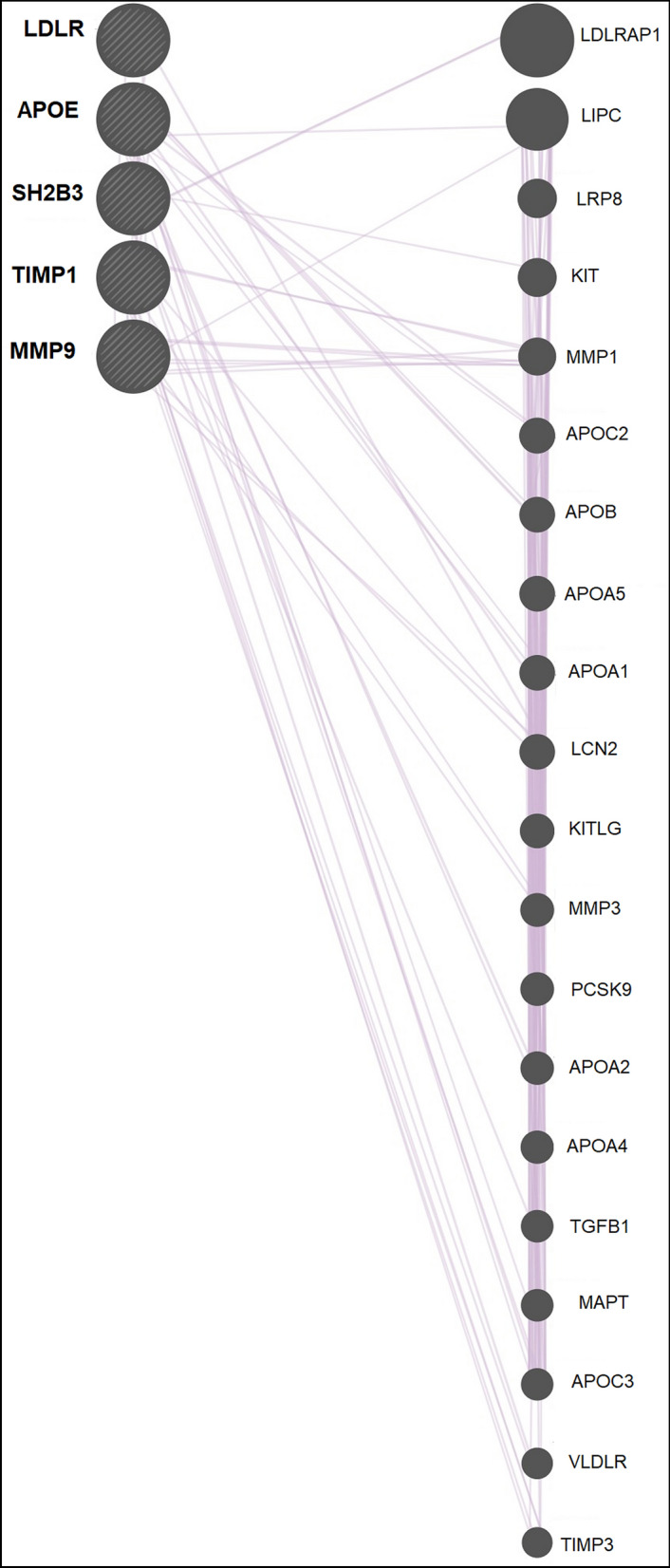
Linear bipartite by GeneMANIA represents Co-expression networks of multi gene queries for *APOE*, *LDLR*, *MMP9*, *SH2B3* and *TIMP1.*

GeneMANIA co-expression network revealed that *APOE*, *LDLR*, *MMP9*, *SH2B3* and *TIMP1 *genes might regulate the expression of several other genes. Single gene queries have shown that *APOE* gene influencing the expression of *APOC3*, *APOA1*, *APOB*, *LIPC*; *LDLR* influences *LCN2*, *TIMP1*; *MMP9* influences *LIPC*, *MMP1*, *LCN2*; *SH2B3* influences *VLDLR*, *LDLRAP1*, *TGFB1*, *KIT*; *TIMP1* influences *VLDLR*, *LDLR*, *MMP1*, *MMP9*, *MMP3*, *LCN2*, *SH2B3* genes (Fig. 4A–E). While multi gene queries interestingly displayed that *APOE*, *LDLR*, *MMP9*, *SH2B3* and *TIMP1 *genes expression are associated with each other (Fig. 5).GeneMANIA consolidated networks revealed that the *APOE*, *LDLR*, *MMP9*, *SH2B3*, *TIMP1* genes are involved in various signaling pathways. It has been shown that *APOE & LDLR* genes are involved in lipid and lipoprotein metabolisms, while *MMP9* and *TIMP1* genes are significantly modulating the degradation of extracellular matrix. In addition, these genes show an internal correlation in their co-expression network (Supplementary Fig. 1).

### Gene ontology enrichment analysis

**Figure 6:**
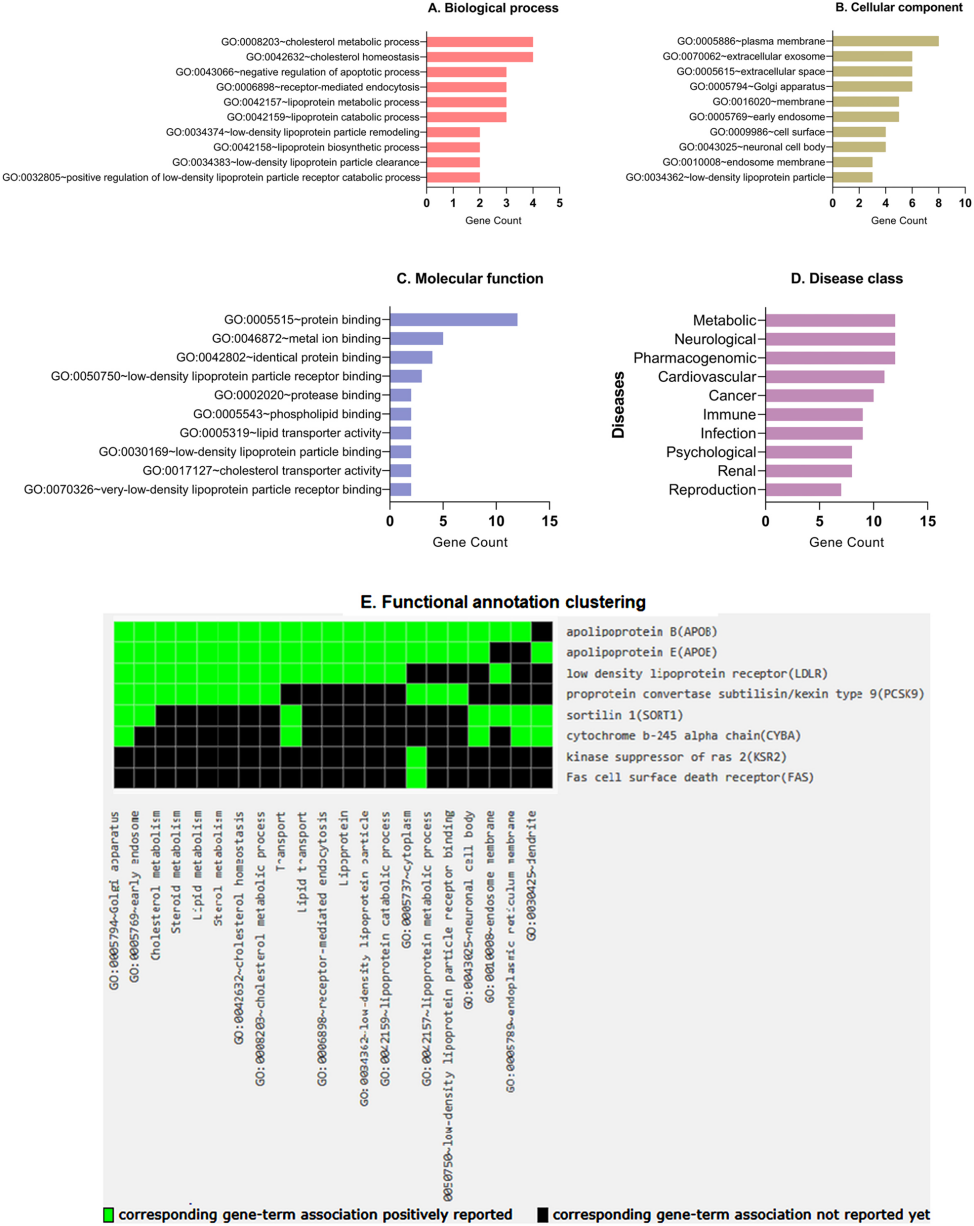
Gene ontology (GO) annotation. The top 10 GO terms in each category. (**A**) Biological process. (**B**) Cellular component. (**C**) Molecular function. (**D**) Disease class. (**E**) Functional annotation clustering.

The gene ontology enrichment analysis of the genes set is shown in Fig. 6. The top 10 GO terms of biological process (BP), cellular component (CC), molecular function (MF) and disease class analyses in genes were sorted by p‑value or gene count. According to the BP analysis, the GO term pathways were mainly associated with the cholesterol biosynthesis, metabolism and homeostasis, regulation of apoptosis, receptor mediated endocytosis, etc (Fig. 6A). For the CC analysis, the GO terms of these genes were mainly located and enriched in the plasma membrane, extracellular exosomes and space, golgi apparatus, etc (Fig. 6B). In the MF analysis, 15 genes were mainly enriched and associated with binding activity and transporter activity particularly protein binding, metal ion binding, identical protein binding, low-density lipoprotein particle receptor binding, cholesterol transporter activity, etc (Fig. 6C).

The GO terms disease class analysis of these genes revealed that the genes are associated with metabolic diseases, neurological diseases, cardiovascular diseases, cancers, etc (Fig. 6D). Later, functional annotation clustering of these genes was performed and functional chart of cluster with highest gene enrichment score (3.17) is shown in Fig. 6E. Out of the 15 genes *APOB*, *APOE*, *LDLR*, *PCSK9*, *SORT1* genes are associated with golgi complex, early endosome, cholesterol metabolism, etc (Supplementary data 1).

## Discussion

The multifactorial diseases like diabetes mellitus, coronary artery disease and cancers are leading cause for morbidity and mortality worldwide. Genetic and epigenetic modifications are also recognized as significant risk factors for the pathophysiology of these diseases. Studies reported that epigenetic modifications play a crucial role in cell differentiation at embryonic development^79^. Besides, environmental factors and age affect the DNA methylation and demethylation patterns in mammalians^80^. The methylation patterns of promoter DNA depends upon the presence of CpG sites, CpG islands existence and their respective size in the promoter region. Genetic variants and epigenetic modifications of CGIs at promoter regions autonomously have a great impact on the regulation of gene expression.

The genes selected for the study are influencing the various pathways such as lipid metabolism and cholesterol homeostasis (*ACAT1*, *APOB*, *APOE*, *LDLR*, *PCSK9*, *SORT1*), oxidative stress (*CYBA*, *KSR2*, *PHOX2A*), apoptosis (*FAS*, *REST*, *SORT1*), inflammation & angiogenesis (*FLT1, SH2B3*), maintenance of extracellular matrix and vascular smooth muscle cells (*MMP9 & TIMP1*). Elucidation of gene expression regulating mechanisms have a significant role in understanding the pathogenesis and risk prediction of several diseases^21–28,30–38,40–51^.

Accumulating evidences have shown that the genetic variants of the *APOE*, *LDLR*, *SH2B3*, *TIMP1*, *MMP9* genes were found to have an impact on risk of the diseases like diabetes, coronary artery disease, acute lymphoblastic leukemia, cancer, lung cancer, etc^21,36,45,52,81–87^.

Dayeh, T. A. et al., have reported that CpG SNVs are associated with differential DNA methylation and gene expression in human pancreatic islets in type 2 diabetics^88^. Hawkins, N. J. et al., and Rapkins, R. W. et al., studied the association of O6-methylguanine-DNA methyltransferase (*MGMT*) gene rs16906252 polymorphism with DNA methylation and reported that the individuals with *MGMT* rs16906252 T-allele has 5.5 folds and 2.64 folds highly methylated than C-allele individuals in colorectal cancer and glioblastoma patients respectively^67,68^. Another study on effect of *RAD50* gene DNase I hypersensitive site7 (RHS7) region rs2240032 polymorphism on DNA methylation has shown that, it is significantly affecting the 5q31 locus *IL13* gene promoter DNA methylation status^69^. To date, there are very limited studies reported on the effect of single nucleotide variations at CpG sites on CpG island existence, size and their respective methylation status.

Furthermore, Palumbo, D. et al., reported that the methylation variability depends upon the CpG cluster density such as high density regions showing low levels of CpG methylation variability, while intermediate density and low density regions have increasingly higher levels of CpG methylation^89^.

Study by Zhou, D. et al., identified 9,42,429 loci for CpG SNPs from HapMap phase II and observed that 51.9% were CpG gain-SNPs and 47.9% were CpG-loss-SNPs and his successive studies on tumor tissues of colon cancer have shown that CpG-loss-SNPs are lowering the methylation in tumor tissues and inferred that the SNPs at CpG sites are significantly associated with traits in cancers^64^. In addition, Wang, Z. et al., identified novel functional CpG-SNPs by conditional false discovery rate (cFDR) analysis from statistical data of two large GWAS of type 2 DM and CAD. Among them, 13 CpG-SNPs of DM, 15 CpG-SNPs of CAD have a significant methylation quantitative trait locus effect and increased susceptibility to disease^65^.

In view of the above, the present study has been designed to analyze the impact of single nucleotide variations at CpG sites in promoter CpG islands of *ACAT1*, *APOB*, *APOE*, *CYBA*, *FAS*, *FLT1*, *KSR2*, *LDLR*, *MMP9*, *PCSK9*, *PHOX2A*, *REST*, *SH2B3*, *SORT1 & TIMP1 *genes on their respective existence and size.

It has been shown that, *APOE* is involved in lipid metabolism, tissue repair, inflammation and plays a significant role in age related diseases. *APOE* modulates its effect on angiogenesis, tumor cell growth and metastasis induction in cancers^90^. A study reported that methylation of *APOE* is significantly lower in men with coronary heart disease than healthy control men and is inversely proportional to *APOE* plasma levels. Thus, it is considered that the DNA methylation is a potential factor for regulation of *APOE* gene expression^19^. In the present study, we have observed that *APOE* rs769448 has abolishing the CGI existence that might influence the methylation pattern and further may regulate the gene expression. The GO enrichment analysis has shown that the *APOE* gene is a key regulator in the cholesterol metabolism and transportation contributing to the initiation and progression of multiple diseases.

Similarly, Low density lipoprotein receptor (*LDLR*) gene encodes a cell surface LDL receptor protein mediating endocytosis of LDL particles regulate cholesterol levels. Evidences suggest that elevated circulating cholesterol levels are involved in the coronary artery disease, cancer growth promotion and progression^91–93^. Ghose, S. et al*.* reported that *LDLR* gene undergoes hypomethylation and induces an increased expression which subsequently decreases the LDL levels and reduces the risk of CAD^94^. In the present study, we have observed that 31% of CpG SNVs abolished the CGI existence and ~ 44% decreased the size of CGI. The abolishment and reduced CGI size, decreases the possibility of methylation and inversely increases the gene expression. The increased gene expression associates with decreased LDL-cholesterol levels and lead to reduced risk of diseases.

Furthermore, Src homology 2-B adaptor protein 3 (*SH2B3*) plays a critical role in haematopoiesis and acts as a negative regulator of several tyrosine kinases and cytokine signaling. *SH2B3* was associated with diseases like atherosclerosis and thrombosis, cancers, diabetes, etc^95–97^. A recent study on Celiac disease (CeD) revealed that the expression of *SH2B3* is influenced by the methylation and it is reported that hypomethylation is associated with higher expression of the genes in CeD patients than controls. The methylated DNA sequence is showing differences in binding of regulatory elements to control the expression of gene at mRNA level^61^. The present study investigations have shown *SH2B3* gene promoter has 7% CGI abolishing SNVs besides 17% size reducing SNVs. The differences in CGI existence, binding of transcription factors and CGI size influences the methylation patterns to regulate the expression. According to gene ontology disease class term *SH2B3* is playing a significant role in metabolic, cardio vascular and immune diseases.

In recent years, there is a growing interest on matrix metalloproteinase (MMP) family to understand their significant association with various disease pathophysiologies such as cancers, CAD and DM^87^. *MMP9* and Tissue inhibitors of metalloproteinases 1 (*TIMP1)* were known to be associated with the risk of cardiovascular disease and several cancers^98–101^. A study on *MMP9* promoter methylation suggested that serum circulating levels were inversely associated with methylation level in Diabetic nephropathy patients. *MMP9* demethylation increases its serum circulating levels that might be accompanying with the incidence and prognosis of diabetic nephropathy^102^. Tissue inhibitors of metalloproteinases (*TIMPs*) are inhibitors of the *MMPs* involved in extracellular matrix degradation. In chronic periodontitis, *TIMP1* promoter methylation positively correlated with severity of the disease^63^. In another study, DNA methylation in *TIMP3* gene contributed to its lower expression and eventually lead to metastasis of oral cancer^103^. In the present analysis, ~ 18% of *MMP9* and ~ 67% of *TIMP1* CpG SNVs have shown for the loss of CGIs, further 57% of *MMP9* and 33% of *TIMP1* CpG SNVs reduced the size of CGI. GO enrichment analysis of *MMP9*, *TIMP1* revealed that these two genes are playing a significant role in metabolic, neurological, cardiovascular diseases and cancers. Altogether, abolishment and reduction of CGI size, differential binding of TFs could influence their gene expression in ECM remodelling and degradation which can further mediate the pathological conditions of various diseases.

Further, 50% of *ACAT1*, ~ 67% of *APOB*, 57% of *CYBA*, ~ 92% of *FAS*, 50% of *FLT1*, ~ 13% of *KSR2*, ~ 44% of *LDLR*, ~ 36% of *MMP9*, 50% of *PCSK9*, 36% of *PHOX2A*, 40% of *REST*, ~ 14% of *SH2B3*, ~ 13% of *SORT1* and 33% of *TIMP1* SNVs are altering the size of CGIs. Among all the 200 SNVs in the genes under study, we have observed that approximately 9% of SNVs at CpG site are abolishing the existence of CpG island; whereas 35% are decreasing the size of CGIs. Consequently, loss of CGI & decreased CGI size leads to the intermittent and asymmetrical DNA methylation pattern of gene which can regulate the expression of genes by affecting binding of transcription factors to the promoter.

The findings of the study suggest that the SNVs at CpG sites in the promoter region regulating CGI existence and size might influence the DNA methylation status and expression of genes that take part in molecular pathways associated with multifactorial diseases like diabetes mellitus, cardiovascular diseases, cancers, etc. The insights of the present study may pave the way for new experimental studies to undertake challenges in DNA methylation, gene expression and protein assays.

## Limitations

A primary limitation of the study is that this is an in silico study, designed to know the impact of single nucleotide variations at CpG sites on CpG island existence, size and their respective DNA methylation pattern and gene expression. Another limitation of the study is that the genes are randomly selected from the various pathways to test the hypothesis. Therefore, the predicted results should be essentially validated using experimental analyses such as genotyping, DNA methylation and their subsequent gene expression assays for further correlation with disease phenotypes.

## Supplementary Information

Supplementary Data 1.

Supplementary Figure 1.

Supplementary Table 1.

## Data Availability

The datasets generated during and/or analysed during the current study are available from the corresponding author on reasonable request.
