# Supplementary material for: In silico identification of single nucleotide variations at CpG sites regulating CpG island existence and size
Source: Sci Rep. 2022 Mar 4;12:3574. doi: 10.1038/s41598-022-05198-8 (PMC8897451; doi:10.1038/s41598-022-05198-8)
Supplement: Supplementary file 2 — Supplementary Figure 1. [file 41598_2022_5198_MOESM2_ESM.docx]

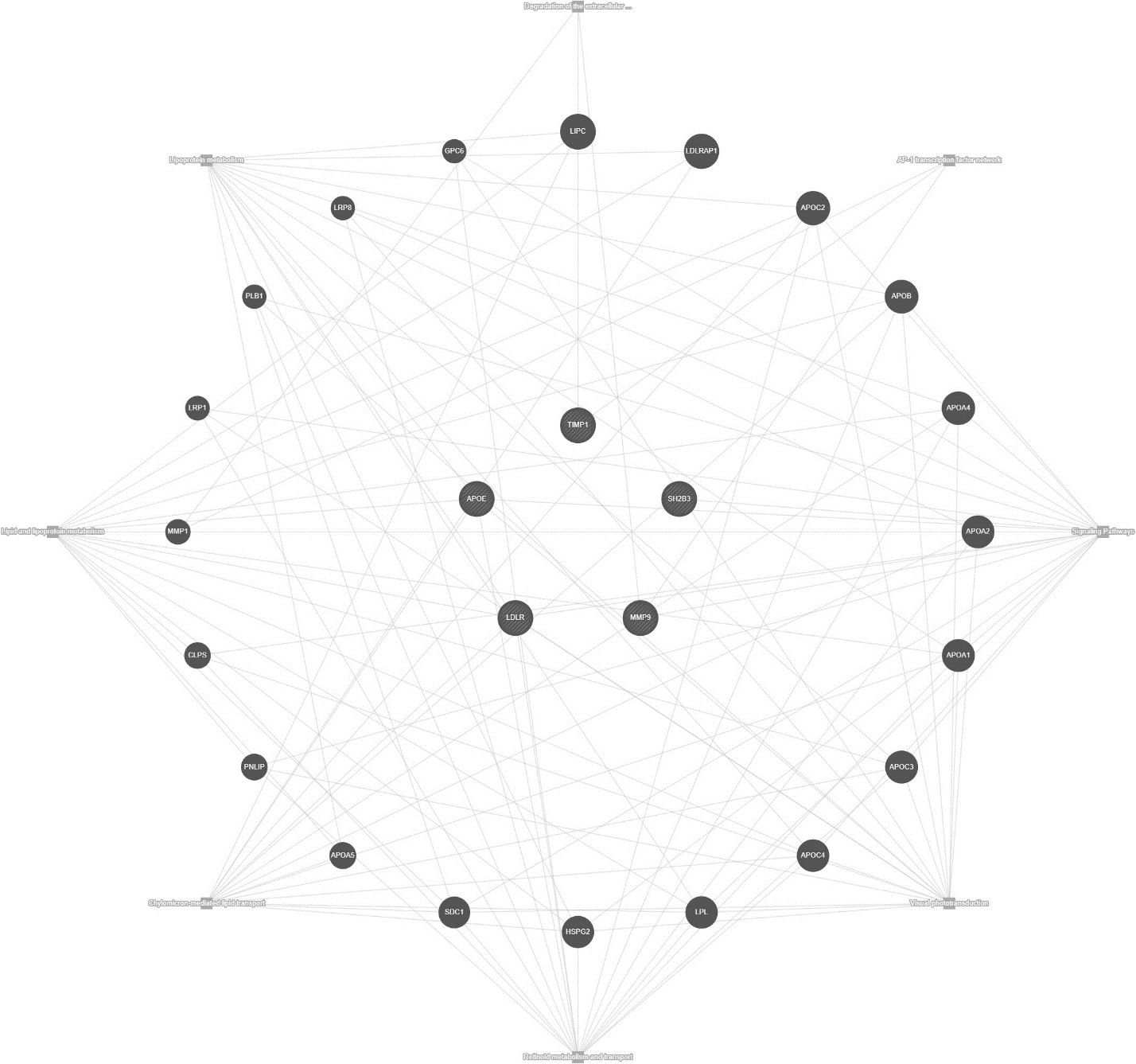
 Supplementary Figure 1. GeneMANIA consolidated pathways for *APOE, LDLR, MMP9, SH2B3* and *TIMP1* genes. *MMP9, TIMP1* genes are involving in the activity (activation/ degradation) of matrix metalloproteinases. *APOE, LDLR & SH2B3* genes are involving in the cholesterol metabolism.
