# Supplementary material for: In silico identification of single nucleotide variations at CpG sites regulating CpG island existence and size
Source: Sci Rep. 2022 Mar 4;12:3574. doi: 10.1038/s41598-022-05198-8 (PMC8897451; doi:10.1038/s41598-022-05198-8)
Supplement: Supplementary file 3 — Supplementary Table 1. [file 41598_2022_5198_MOESM3_ESM.doc]

Supplementary Table 1. List of selected genes involved in various pathways in DM, CAD & cancer and study populations

| **S No** | **Name of the Gene** | **Role/ Pathway of the gene** | **Author name** | **Study population/**  **Type of study** |
| --- | --- | --- | --- | --- |
|  | ***ACAT1*** | Cholesterol homeostasis | Wang, Y. T. *et al 2017* | Chinese |
|  |  |  | Zabielska, J., et al., 2019 | Review |
|  | ***APOB*** | Major apolipoprotein of LDL in coronary heart disease | Ya Yun Feng et al., 2020 | Meta-analysis |
|  |  | Lipid metabolism | Hayat, M. et al. 2020 | African |
|  | ***APOE*** | Lipid metabolism | Sudong Liu et al., 2019 | Chinese |
|  |  |  | Canwang Wang et al., 2019 | Chinese |
|  |  |  | Mostafa Saadat 2012 | Meta-analysis |
|  | ***CYBA*** | Oxidative stress in Breast cancer and coronary artery disease | Tupurani, M. A. et al. 2018 | Asian Indian* |
|  |  |  | Nowak, T. *et al 2018* | Polish Caucasians |
|  | ***FAS*** | Apoptosis in coronary artery disease & Pancreatic β-cells | Kumar, G. K. *et al. 2016* | Asian Indian* |
|  |  |  | Anuradha, R., 2014 | Review* |
|  | ***FLT1*** | Angiogenesis &  Inflammation | Kurotsu, S. *et al. 2018* | In vitro |
|  |  |  | Qian, B. Z. et al., 2015 | In vivo |
|  | ***KSR 2*** | Ras-mediated tumorigenesis | Neilsen, B. K., 2017 | Review |
|  | ***LDLR*** | Lipid metabolism | Hayat, M. et al. 2020 | African populations |
|  |  |  | Do, R., et al., 2014 | NHLBI Exome Sequencing Project- (11 studies) |
|  | ***MMP9*** | Extracellular matrix degradation in Breast cancer, coronary artery disease & Type 2 diabetes | Padala, C. *et al. 2017* | Asian Indian* |
|  |  |  | Hassanzadeh-Makoui, R., et al., *2020* | A systematic review and Meta-analysis |
|  |  |  | Singh, Kanhaiya et al., 2013 | Asian Indian |
|  | ***PCSK9*** | Regulation of LDL receptor recycling | Benn, M. et al., 2010 | 3 Independent Studies and Meta-Analyses |
|  |  | affect β-cell insulin secretion | Kockx, M. & Kritharides, L. 2019 | Editorial Commentary |
|  |  | Promoter DNA methylation | Nivas S et al., 2021 | Asian Indian* |
|  | ***PHOX*** | Oxidative stress | Andreas Gardemann et al., 1999 | Caucasians |
|  |  |  | Tao Li et al., 2015 | Meta-analysis |
|  |  |  | Stephanie Anais Castaldo et al., 2015 | Portuguese |
|  | ***REST*** | Anti-apoptosis & transcriptional repressor | Yu, Y. et al., 2012 | Chinese |
|  |  |  | Thomas F. Westbrook et al., 2005 | *In vitro* |
|  | ***SH2B3*** | Inflammatory pathway & hematopoiesis | Lu Hong et al., 2018 | Meta-analysis |
|  |  |  | Yan Gu et al., 2018 | Chinese |
|  |  |  | Lavrikova, E. Y. et al 2011 | Russian |
|  | ***SORT1*** | Cholesterol homeostasis & Apoptosis | Kiran Musunuru et al., 2010 | Swedish |
|  |  |  | Federico Biscetti et al., 2019 | Italy |
|  |  |  | Roselli, S. et al. 2015 | Australian |
|  | ***TIMP1*** | Inhibitors of the matrix metalloproteinases (MMPs) & tumor cell apoptosis | ChunyanMeng et al., 2018 | Meta-analysis |
|  |  |  | Guangcun Cheng et al., 2016 | Human breast cancer cell lines |

*studies published from our laboratory
